# Nonmechanical varifocal metalens using nematic liquid crystal

**DOI:** 10.1515/nanoph-2023-0001

**Published:** 2023-02-28

**Authors:** Shuangqi Zhu, Qiang Jiang, Yongtian Wang, Lingling Huang

**Affiliations:** School of Optics and Photonics, Beijing Engineering Research Center of Mixed Reality and Advanced Display, Beijing Institute of Technology, Beijing, 100081, China

**Keywords:** liquid crystal, metasurface, Moiré lens, nonmechanical, varifocal

## Abstract

Metalenses exhibit a substantial potential in replacing traditional optical component as they present a methodology for miniaturization. Lenses with tunable focal lengths can play a key role in various fields with applications in imaging, displays, and augmented and virtual reality devices. Here, we propose an electrically controllable varifocal metalens at the wavelength of 950 nm. The metasurface cascaded with nematic liquid crystal is integrated into an analog chip, which providing sequential specific two-dimensional addressable voltage patterns. The focal length of the reflective light can be modulated continuously with the change of voltage patterns. For the super-pixel cell with 6 μm period at a low voltage of 6 V, the zoom range and the zoom ratio are demonstrated to be 180 mm and 34, respectively. Besides, attributing to the enhanced forward scattering of Huygens metasurface and the large birefringence index of the liquid crystal, along with the integrated circuit compatible design, our metalens owns high integration in the NIR band under considering the practical processing. Therefore, the proposed nonmechanical varifocal metalens may unleash the full potential of continuous zoom metalens for micro-optical display and imaging applications in the future.

## 1 Introduction

Modern handheld devices such as smartphones typically include a miniaturized camera. Different approaches have been utilized for varifocal lenses systems. One of the most traditional ways is to change the relative axial distance between the composition lenses. However, even by using deformable mirrors to reduce the light path, the thick and bulky traditional optical components are still inevitable. Another way is to use the liquid lens [1–3], but this method has limited application prospects for high applied voltage. Hence, in general, optical zoom utilizing the lens system usually suffers from the large volume. In addition, the rapid growth of augmented reality and virtual reality (AR/VR) is triggering a corresponding boom in the demand for micro-optical systems. As a result, there is an urgent need for miniaturized continuously varifocal optical system.

Metasurface is a two-dimensional array of metal/dielectric antennas with spatially varying phase response and subwavelength separation [4–17]. Through delicate design of the antennas, the metasurface can imprint arbitrary phase distributions on propagating light. Metalens can minimize the volume of imaging systems tens or even hundreds of times, making the whole device ultra-thin and more compact [18–27]. Different varifocal optical systems have been proposed by using metalenses. For instance, to use dynamic materials, such as phase change material or the liquid crystal (LC), but the research based on this method can only realizing binary change of the focal length to date [28, 29]. In terms of the continuous zooming, the metalens can be realized by stretching some elastomeric materials, but with limitations of nonimmediate response, spherical aberration, and distortion from nonideal spherical profiles [30, 31]. In addition, it can be barely used in a vertical direction due to the gravity effects. With the help of Micro-Electro-Mechanical System (MEMS), the focal length can also be tuned continuously [32, 33]. Two lenses are usually included in the system and one piece is translated along the axis or in the plane or rotated relative to the other. Among them, the rotation transformation is the basic zoom principle of the Moiré lens [34–39], which consists of two phase plates with complementary phase profiles. By changing the mutual rotation angle between them, the focal length changes accordingly. However, the approaches mentioned above still have room to improve, for they all need to mechanically adjust the components to change the focal length, which will incur additional rotation error and system instability.

In this work, we propose an electrically controllable varifocal metalens based on the principle of the Moiré lens in near infrared region. The phase of Moiré lens is reconfigurable when providing sequential specific two-dimensional addressable voltage patterns onto an analog liquid crystal chip. With the integrated circuit compatible design, the Huygens metasurface cascaded with the nematic LC enables our metalens featuring in the zoom range of 180 mm and the zoom ratio of 34 for the super-pixel cell with 6 μm period at a low voltage of 6 V. The average efficiency can reach 32.3% under considering the practical processing, which is comparable with the relevant works. The proposed continuous varifocal metalens is expected to shrink the optical systems in display and imaging applications, for instance, the AR/VR system, smartphones, endoscope, and so on.

## 2 Results

**Figure 1:**
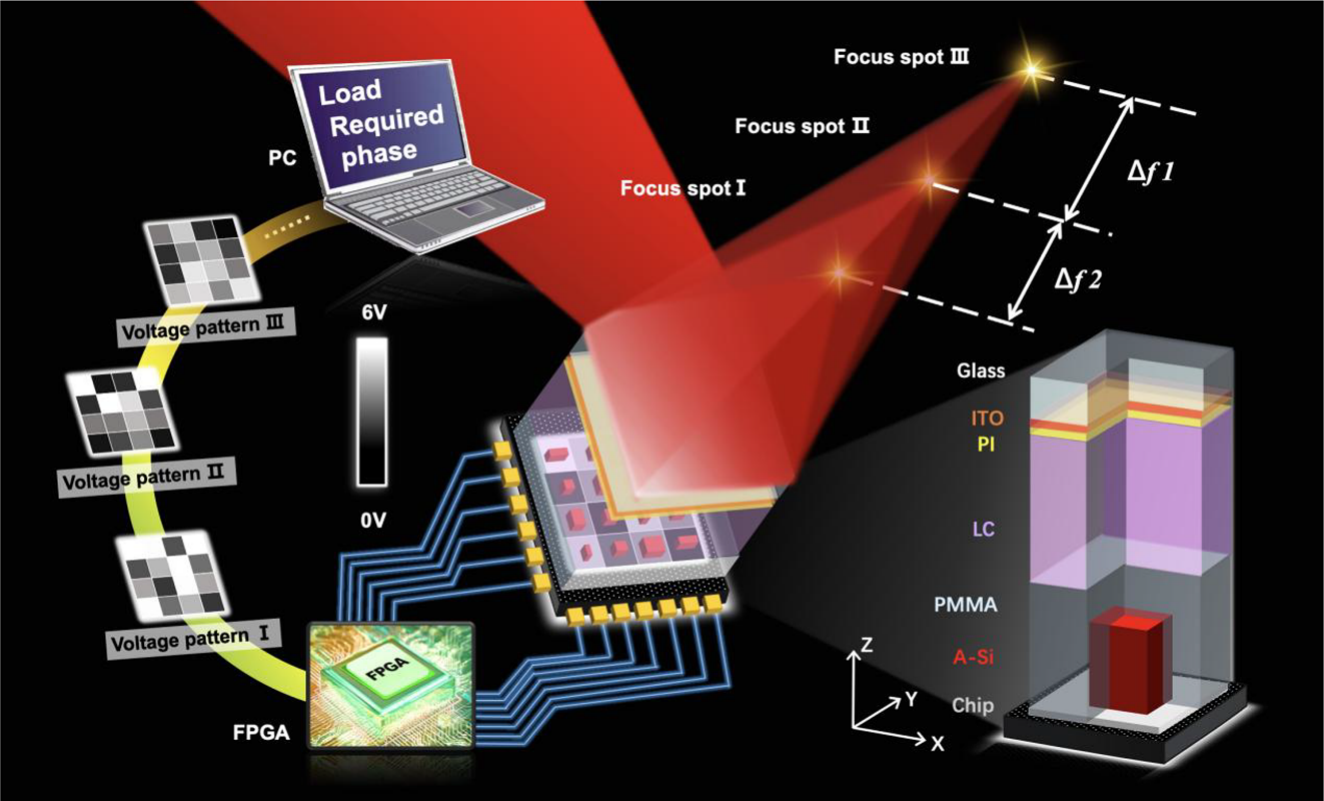
Schematic illustration of the LC nonmechanical varifocal metalens. The metasurface coated by PMMA and the electrically controllable LC layer function as two complementary phase plates for the Moiré lens. By loading different two-dimensional voltage patterns on the analog chip, the focal length of the reflected light can be adjusted continuously.

The proposed electrically controllable varifocal metalens that composed of nematic LC and metasurface is inspired by the Moiré lens. As is illustrated in Figure 1, the Huygens metasurface protected via a thin PMMA layer is cascaded with nematic LC and then integrated into an analog chip. By exerting specific two-dimensional addressable voltage pattern in the electrode array of the analog circuit, the refractive index distribution of the nematic LC changes, yielding another desired phase distribution for varifocal metalens based on Moiré effect. The traditional Moiré lens requires changes mechanically rotation for varifocal function, while in our design, the focal length of the reflective light can be modulated continuously by continuously changing voltage without any mechanical adjustment.

**Figure 2:**
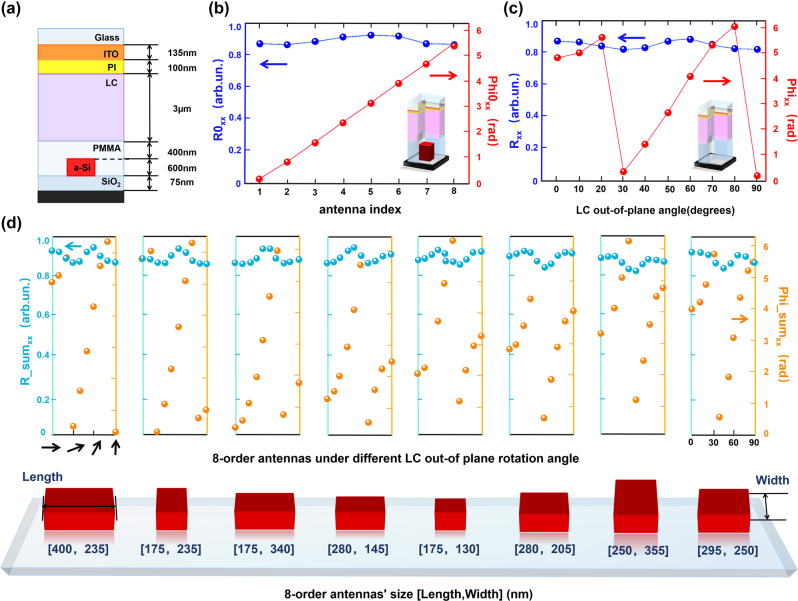
Simulation results of the dielectric antenna. (a) The schematic diagram of the unit cell. (b) The reflection coefficient (amplitude *R*0_*xx*_ and phase *Phi*0_*xx*_) of the first pixel model with only selected 8-order antennas. Both the LC’s in-plane and out-of-plane angle are fixed at 0° under *x*-polarized illumination. (c) The reflection coefficient (amplitude *R*_*xx*_ and phase *Phi*_*xx*_) of the second pixel model with the antenna removed and only the LC out-of-plane angle changes from 0° to 90° under *x*-polarized illumination. (d) After reassembling the two pixel models, each 8-order selected antenna maintains a high reflectivity and covers the range from 0 to 2π when the LC out-of-plane angle changes from 0 to 90°.

The unit of such varifocal metalens is composed of antenna, PMMA, and liquid crystal fabricated on the voltage-addressable chip. Figure 2(a) shows the side view of the metalens pixel whose period is *P* = 500 nm. The bottom analog chip is composed of addressable two-dimensional units, on which a SiO_2_ layer of 75 nm is deposited as a protective layer. The amorphous silicon antennas (*H* = 600 nm) are used for designing metalens operates in the near infrared (NIR). A 1-μm-thick insulative PMMA layer is introduced as the isolation layer to minimize the interaction between the LC and the antenna. The selection criteria for insulation thickness takes the voltage division of the LC layer into account, which is further expanded in the Discussion part. The thickness of the nematic LC E7 layer is 3 μm. The upper layer of the cell is a conductive ITO glass (ITO’s thickness is 135 nm) that coated with the LC orientation layer (polyimides, PI) with the thickness of 100 nm, initially orienting the LC molecules along the *X*-axis. Here, we define in advance the in-plane angle and the out-of-plane angle of the LC with respect to the *XY* and *XZ* plane, respectively.

Our design is based on the principle of the Moiré lens, which consists of two paired phase plates. By adjusting the mutual angle between them, one can change the focal length continuously. The phase distribution of one phase plate can be written as [37]

(1) $$\Phi \left(r,\theta_{0}\right)=round\left(\frac{1}{\lambda \cdot F_{0}}r^{2}\right)\theta_{0}$$

where *λ* is the operation wavelength, *F*_0_ represents the reference focal length, and it is set as 45 μm for the pixel with period of 500 nm. *r* is the radial coordinate on the metasurface. *θ*_0_ denotes the reference rotation angle and is set as 0 for simplicity The round (•) function transforms the value of the operand to the nearest integer to avoid the sectoring effect. The phase distribution of another phase plate is complementary to the above one. Thus, the phase of the modulated light passing through the paired plates can be described as

(2) $$\Phi_{\text{integral}}=\Phi \left(r,\theta_{0}\right)+\left(-\Phi \left(r,\theta_{0}-\theta \right)\right)=ar^{2}\theta$$

where *a* is a constant and *θ* denotes the relative angle between the paired phase plates. To realize the phase requirement of the focus lens, *a* can be adopted as 1/*λF*_0_. The relationship between the tunable focal length (*f*_*θ*_) and relative angle (*θ*) can be written as

(3) $$f_{\theta }=\pi /a\theta \lambda$$

Thus, the phase patterns of two phase plates of the Moiré lens can be obtained from Eq. (1) and (2).

In our design, we separate the metasurface and the LC layer as two gapless phase plates of the Moiré lens. The electrical modulation to the LC layer can refresh the phase profile with different rotations. In simulation, we build two different pixel models and sweep their parameters, respectively. In the first pixel model, both the in-plane and out-of-plane angle of the LC are fixed at 0°, and we only sweep the antenna’s parameters (length and width) with RCWA method to obtain the reflection coefficient (contains the amplitude and phase information of reflected light) under the illumination of *x*-polarized light at *λ* = 950 nm. From all the sweeping results, eight antennas with high reflectivity and capable of a 2π phase range was used to build a metasurface with an eight-level phase distribution. Figure 2(b) shows the amplitude *R*0_*xx*_ and phase *Phi*0_*xx*_ of the eight selected antennas under the first pixel model setting, which is used to match the phase distribution of the Moiré lens’s first phase plate.

In the second pixel model, the antenna was removed, and the in-plane-angle of LC is still fixed at 0°. Only the out-of-plane angle of LC molecules changes from 0° to 90° with a step of 10° in the sweeping operation. As shown in Figure 2(c), the reflection coefficient (amplitude *R*_*xx*_ and phase *Phi*_*xx*_) under different LC out-of-plane angles can also cover a 2π range along with high reflectivity, which is used to match the phase distribution of the Moiré lens’s second phase plate. To further verify whether the current parameters sufficient enough to build the final varifocal metalens, we simulate each of the 8-order antennas under all possible 10-order LC orientations, that is, reassembling the two pixel models. As shown in Figure 2(d), for different 8-order antenna size we selected before, one can enable the reflective light to satisfy the high reflection coefficient and 2π phase coverage in a single-period pixel by both controlling the antenna’s size and the LC out-of-plane angle.

**Figure 3:**
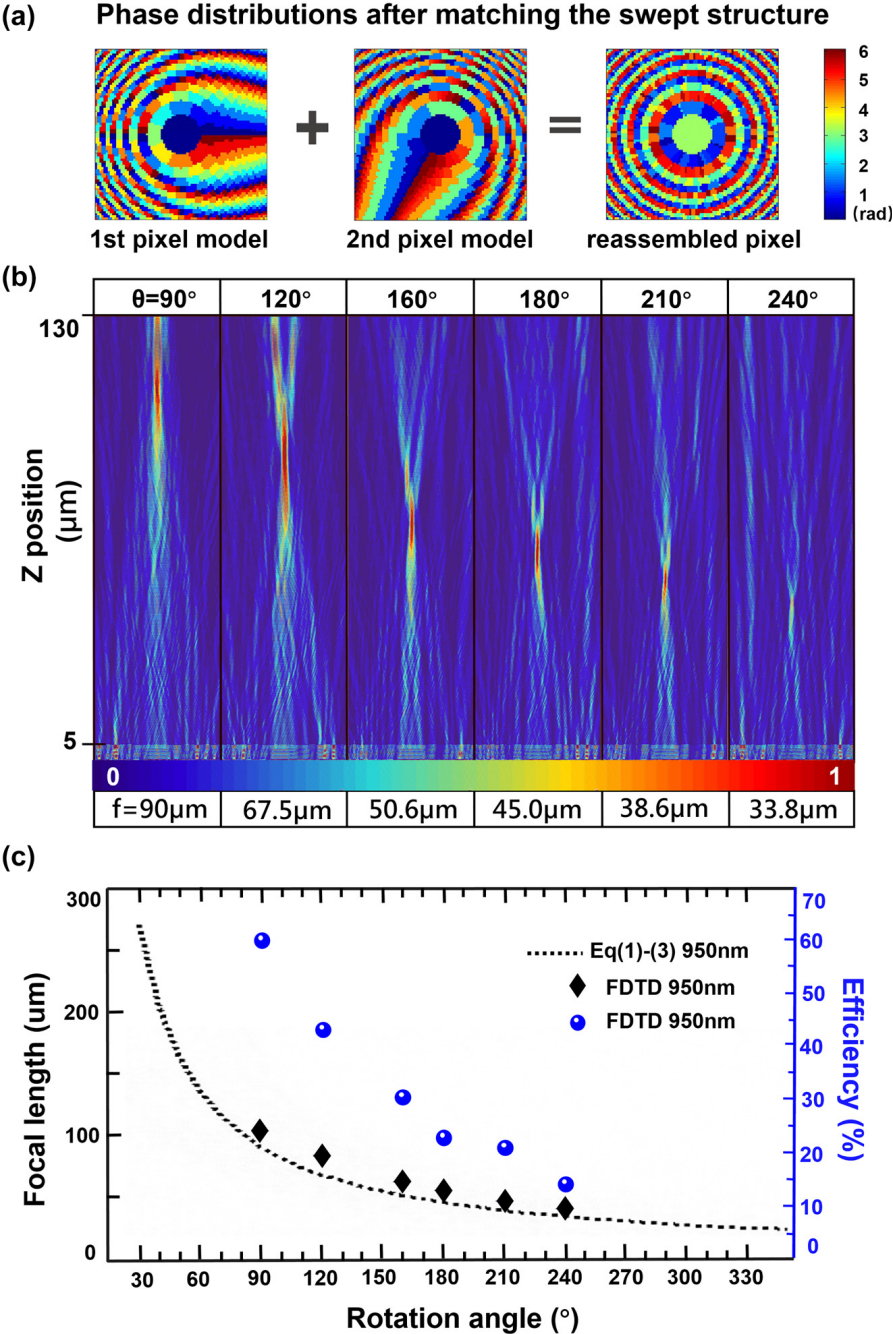
Simulation results of the proposed varifocal metalens. (a) The matched phase distribution. The left image corresponds to the first pixel model/the metasurface layer. The middle image corresponds to the second pixel model/the LC layer. The right image corresponds to the integrated device. (b) Focusing results of the metalens under different relative rotation angle *θ* of the Moiré lens (*θ* = 90, 120, 160, 180, 210, 240°) at *XZ*-plane. (c) The comparison graph between the theoretical and simulation results, and the calculated focus efficiency.

When setting the relative rotation angle *θ* between the Moiré lens’s two phase plates at 120°, the corresponding matched phase distribution result is shown in Figure 3(a). We utilize the antennas’ size distribution **S** and the LC’s out-of-plane angle distribution **A**_120°_ to create a varifocal metalens with 81 × 81 integrated pixels. The final matched phase distribution result exhibits the typical ring-shaped phase distribution of a focusing lens. Using the same method, we obtained the corresponding metalens settings under other relative rotation angle of the Moiré lens (*θ* = 90,160,180,210,240°), that is, **S** remains fixed like the Moiré lens’s first plate and **A** keeps changing to obtain **A**_90°_/**A**_160°_/**A**_180°_/**A**_210°_/**A**_240°_, which is equal to mechanically rotate the Moiré lens’s second plate. Then we extract the data from the middle line in **A** and **S** to form the one-dimensional metalens consisting of 1 × 81 pixels and do the full wave simulation by using finite difference time domain method. In Figure 3(b), the varifocal performance of the proposed metalens can be clearly seen at the *XZ* plane. Then we compared the focal length results obtained from the simulation to that from the theoretical calculation.

According to the Eqs. (1)–(3), the focal length can vary from 405 μm to 23 μm (zoom range ≈0.38 mm) with *θ* changes from 20 to 350° at *λ* = 950 nm theoretically, the partial result (30°< *θ* < 350°) is shown as the dotted line in Figure 3(c). Meanwhile, the focal lengths of the six **A** sets implemented in the FDTD are shown as a function of the rotation angle *θ*, which is in qualitative agreement with theoretical predictions. But at each *θ*, the simulation results are always higher than that of the theoretical results due to large depth of focus. Figure 3(c) also shows the focusing efficiency of our metalens, which is defined as the fraction of the incident light that passes through a circular iris on the focal plane with a radius equal to three times the FWHM spot size [40, 41]. The average focus efficiency can reach 32.3% and the obtained results satisfy the Moiré lenses’ inherent efficiency variation trend, namely, the focusing efficiencies decrease when rotation angle increases. That is because of the periodicity of the transmission function of Moiré lens, the effective area of the combined lens would decrease when rotation angle increases. Besides, there is background noise in the intensity distribution at *XZ* plane, especially when the *θ* reaches 240°, the noise is greater. This may be due to the small number of pixels used in the simulation and we believe the noise could be decreased by using more pixels with more powerful computing resources.

**Figure 4:**
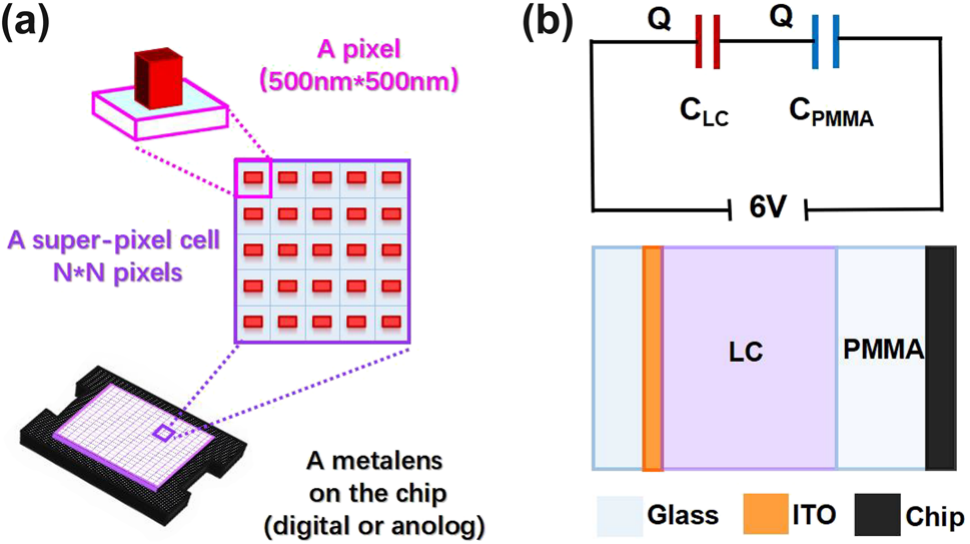
Schematic illustration of the super-pixel cell layout and the equivalent capacitance model. (a) Schematic of the super-pixel cell and the integrated configuration of the metalens. (b) Schematic of the equivalent capacitance model for analyzing the insulation layer’s thickness.

To avoid the neighboring coupling between different liquid crystal pixels, we propose the concept of the super-pixel cell that is replacing a single pixel with *P* = 500 nm to a two-dimensional super-pixel cell containing *N* × *N* identical antennas as shown in Figure 4(a), where *N* = 5 is shown as an example. This pixel setting can minimize the crosstalk caused by the inherent influence between LC molecules in adjacent pixels and guarantee the metalens achieve a finer phase manipulation. We choose an analog chip with a unit size of 6 μm × 6 μm and fabricate 12 × 12 identical antennas in each super-pixel cell. That is assuming a large area metalens composed of 151 × 151 super-pixel cells on the analog chip with a diameter D of 0.906 mm. Considering that *F*_0_ is a variable in the Moiré lens’ equation, the size of the metasurface, the better focusing effect under large relative rotation angle *θ*, and a reasonable numerical aperture (NA) setting in practical applications, the zoom range of our metalens is a result of multiple factors. After taking all the influencing factors into account, we set *F*_0_ = 10.5 mm for super-pixel cell *p* = 6 μm. According to Eqs. (1)–(3) and supported by our simulation results, its focal length can be tuned from 5.4 mm to 189 mm when the **A** changes from **A**_350°_ to **A**_10°_. We further define the zoom ratio (ZR) to better describe the zoom capability of our device, which is calculated by the following equation:

(4) $$ZR=\frac{f_{\text{initial}}+\Delta f}{f_{\text{initial}}}$$

where *f*_initial_ is the shortest focal length and Δ*f* is the zoom range. Namely, ZR is the ratio of the longest focal length to the shortest focal length and the corresponding result are ZR = 34. It also should be noted that although only the simulation results are shown in the paper, the parameter design of the metalens is considered to meet the actual processing needs.

Besides, by using commercial processing of liquid crystal package instead of manual assembly, our device will be further miniaturized through reducing the LC and the PMMA insulation layers, or can realize directly fabricate the metasurface immersed in the LC layer without the PMMA spacer layer so that the ultrathin nature of metasurface can relieve the thickness constraint of the conventional LC on silicon (LCoS) devices and boost the response speed of metasurface-integrated LCoS devices.

## 3 Discussion

### 3.1 The insulation layer

There is a transparent PMMA layer in metalens to prevent the LC from contacting the antennas. Due to the PMMA’s insulativity, its thickness should be considered carefully especially in electrical-controlled device. Here, we set the thickness of PMMA to 1 μm after balancing the insulation and actual fabrication. We choose amorphous silicon as antenna material because of its good conductive properties. Nevertheless, we ignore it here for considering the extreme insulated case in the following theoretical model. The thin PI layer and SiO_2_ layer are also ignored. We simplify the whole device as a series capacitor circuit and regard each layer as a flat capacitor with the equal charge *Q*. The voltage loaded on the analog chip is generally 6 V and that on the LC layer should be at least 3 V to achieve the sufficient phase modulation. The schematic of the capacitance model is shown in Figure 4(b). Considering the series capacitor circuit, we can obtain the following formula:

(5) $$U_{\text{LC}}+U_{\text{PMMA}}=U_{\text{total}}=6\;V$$

(6) C=εS/d=Q/U

(7) $$\begin{array}{ll} \frac{U_{\text{LC}}}{U_{\text{PMMA}}} & =\frac{C_{\text{PMMA}}}{C_{\text{LC}}}=\frac{\varepsilon_{\text{PMMA}}\cdot S\;/d_{\text{PMMA}}}{\varepsilon_{\text{LC}}\cdot S\;/d_{\text{LC}}}\approx \frac{1}{d_{\text{PMMA}}}\left(\frac{\varepsilon_{\text{PMMA}}d_{\text{LC}}}{\varepsilon_{\text{LC}}}\right) \end{array}$$

where *ε* is the dielectric constant of the medium between the upper and lower plates, *S* is the plate area, and *d* corresponds to the thickness of each layer in our device. According to Eq. (6), the partial voltage ratio of the LC and PMMA layers is related to the thickness of the PMMA layer, for the value in the right parentheses is a constant at the fixed working wavelength. After calculation, we find that the LC layer can divide the voltage near 4 V even when the insulating layer thickness reaches 1 µm. Thus, we finally set the *d*_PMMA_ = 1 μm, which guarantee that enough voltages for the LC layer in the extreme case and high feasibility of the fabrication. However, considering the actual processing difficulty, the thickness of PMMA can shrink to 0.6 µm or less, resulting in thinner devices.

### 3.2 The LC layer

In this paper, the thickness of the LC layer is fixed as 3 μm, which is the best value we can reach to guarantee the good hand-processed yield until now. Phase coverage of individual liquid crystal layer at different LC rotation angles can be calculated by the following equations:

(8) $$\frac{1}{n_{\text{eff}}^{2}}=\frac{\text{co}s^{2}\theta_{\text{LC}}}{n_{e}^{2}}+\frac{\text{si}n^{2}\theta_{\text{LC}}}{n_{o}^{2}}$$

(9) $$\Gamma =\frac{2\pi d_{\text{LC}}\left(n_{\text{eff}}-n_{o}\right)}{\lambda }$$

where *θ*_LC_ is the LC rotation angle out of the plane, the Γ is the amount of phase delay when light passing through a LC layer with thickness of *d*_LC_. For the reflective device, the phase coverage of 2π can be satisfied theoretically when using 3-μm nematic LC layer at 950 nm (LC E7, *n*_e_ = 1.70, *n*_o_ = 1.51, Δ*n* ≈ 0.2 @950 nm). The thickness of the liquid crystal can be further reduced to ensure faster response time. Even so, the LC thickness of 3 μm is comparable to current cutting-edge research of the LC based varifocal metalens.

## 4 Conclusion

In summary, we propose an electrically controllable varifocal metalens based on the principle of the Moiré lens in near-infrared region. The metasurface cascaded with nematic LC is integrated into an analog chip which providing sequential specific two-dimensional addressable voltage patterns. The focal length of the reflective light can be modulated continuously with the change of voltage patterns. Attributing to the enhanced forward scattering of Huygens metasurface, the large birefringence index of the LC, the selection of amorphous silicon at NIR band, and the integrated circuit compatible design, our metalens enables a zoom range of 180 mm for the super-pixel cell with 6 μm period at a low voltage of 6 V. We simplify the whole device as a series capacitor circuit to calculate the partial voltage of each layer, ensuring the insulation layer and LC layer can be allocated to the appropriate working voltage at the current thickness. Note that all the parameter design of the metalens is considered to meet the actual processing needs. Because our metalens features in good adaption to the mature circuit technique, we expect that by combining with the updated processing technology, such metalens can play a certain role in the next generation of micro-optical display or detection in the future, such as AR, VR, LIDAR, and other vital applications.

## 5 Methods

### 5.1 Numerical simulation

Numerical simulations were done by two steps. First, we use Rigorous Coupled Wave Analysis (RCWA) for antennas’ structural sweeping and selecting. Second, we use the finite-difference time domain for validating the performance of the proposed varifocal metalens.

### 5.2 The simulation in RCWA

The initial orientation of LC is parallel to the *X*-axis and its original diagonal matrix for the “off” voltage can be written as $\varepsilon_{LC}\left(\alpha =0^{◦}\right)=\mathrm{diag}\left(n_{e}^{2},n_{o}^{2},n_{o}^{2}\right)$ . We express the LC permittivity tensor with specific out-of-plane angle *α* by multiplying the corresponding orientation matrix to the original diagonal matrix. Thus, the LC permittivity tensor can be written as $\varepsilon_{\text{LC}}\left(\alpha \right)=R_{y}^{-1}\left(\alpha \right)\cdot \varepsilon_{\text{LC}}\left(\alpha =0^{◦}\right)\cdot R_{y}\left(\alpha \right)$ , where $R_{y}\left(\alpha \right)$ is the rotation matrix about the *Y*-axis. Afterward, the amplitude and phase information of a single pixel with specific antenna size and LC orientation angle can be retrieved through the full-wave simulation.

### 5.3 The simulation in FDTD

For the single meta-atom in one pixel, period boundary conditions (PBCs) are set in both the *X*- and *Y*- directions. The top glass and bottom reflective aluminum layer were modeled as semi-infinite media. The E7 LC was modeled as an anisotropic medium with the material parameters taken from the Ref [42] and the LC orientation is defined by the in-plane azimuthal angle and the polar angle. The former is always equal to 0 in our work and the latter varies by 10° from 0 to 90°. The refractive index and the orientation angle of the LC and the size of the antenna in each pixel needs to be modeled according to the phase patterns. For one-dimensional metalenses, the perfectly matched layers (PMLs) are set in the *X*- direction and PBCs are set in the *Y*- direction. After that, the whole settings are calculated, and the result is recorded by *XZ*-plane monitor.
